# “As the Dew Is Dried Up by the Morning Sun, So Are Mankind’s Sins at the Sight of Himalaya”

**DOI:** 10.3201/eid1406.0608

**Published:** 2008-06

**Authors:** Polyxeni Potter

**Affiliations:** * Centers for Disease Control and Prevention, Atlanta, Georgia, USA

**Keywords:** Art science connection, Nepal, HIV co-infections, thangkas, Nepalese painting, sex-trafficked women and girls, paubhas, Himalaya, Tibet, about the cover

**Figure:**
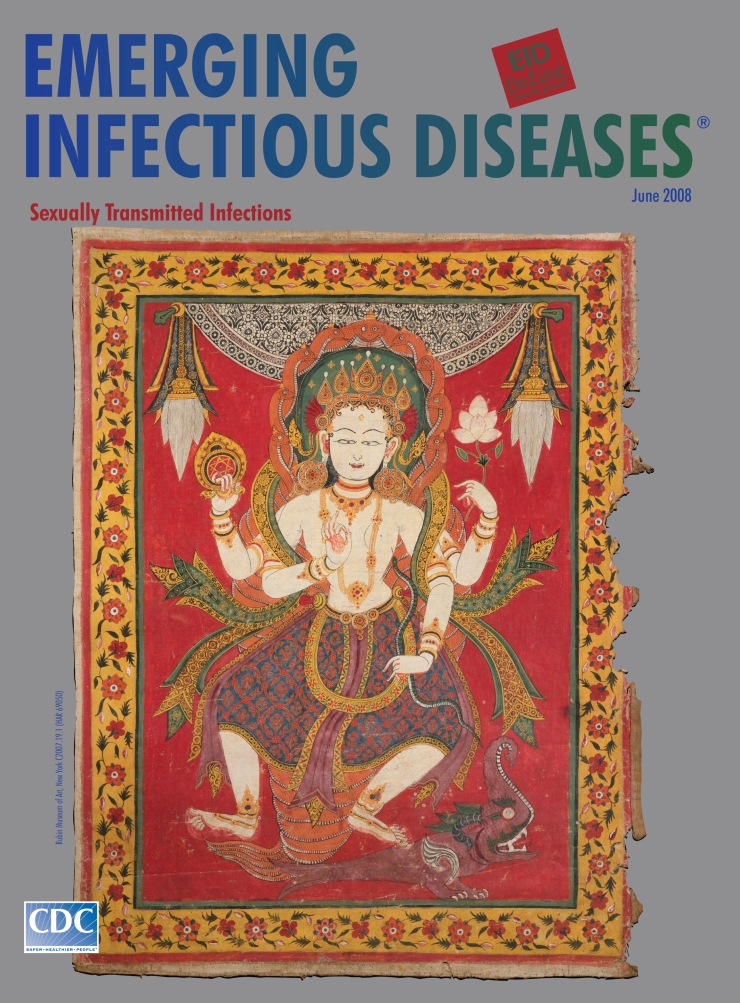
**Festival Banner, Nepal, 17th century.** Ground mineral pigment on cotton (60.325 cm × 44.45 cm). Rubin Museum of Art, New York. C2007.19.1 (HAR 69050)

—The *Puranas*, scriptures of ancient India

The Himalaya, “abode of snow,” dominates the landscape of Nepal. Home of the highest peaks in the world, including Mount Everest, the country is traversed and bound by the massive range. “In a hundred ages of the gods, I could not tell thee of the glories of the Himalaya,” elegize Hindu texts. A barrier to the movement of people for thousands of years, the slopes, glaciers, rivers, and lakes associated with the range discouraged outside interference and military adventures. In the remote gorges, valleys, and high plateaus, flourished instead the individual and unique cultures of a diverse population.

Landlocked in the south by India, the north by Tibet, present-day Nepal historically comprised the kingdoms of the Kathmandu Valley. At the crossroads of trade and multiple religious traditions (Hinduism, Buddhism, Jainism, Sikhism), the valley became a shared sacred site, a focus of social and cultural exchange, and the creative locus of much Himalayan art. The symbiosis of Hinduism and Buddhism is reflected in the art of the indigenous Newar.

When famed Chinese envoy Wang Hsüan-tse visited Nepal in the 7th century, he wrote with admiration of the metalwork in King Narendradeva’s palace in Kathmandu and mentioned the king’s elaborate belt decorated with golden images of Buddha (*1*). He also noted many architectural marvels and houses embellished with sculptures and paintings.

Around the same time, with Mahayana Buddhism new to Tibet having been introduced through Nepal during the reign of Angshuvarma, demand was high for icons and sacred texts. Many, including scriptures referred to as the Prajñāpāramitā, were copied in the Kathmandu Valley. Surviving texts contain images of religious figures. Gifting manuscripts to monasteries or temples was valued as high virtue, so manuscript illumination persisted for centuries (*2*).

The same colorful, theatrical, and detailed style perfected in manuscripts is also found in larger paintings on cloth. These religious paintings, used both to enhance and to record the contemplative experience, were known as *paubha*, the Newari term for the kind of painting called *thangka* in Tibet (*3*). Paubha may have originated with Newar artists who worked in Tibet producing metalwork, murals, and illuminated manuscripts. To meet increased demand for religious scenes, they created a new type of painting on cloth that could be easily rolled up for transport. Paubha may have also originated when Songtsän Gampo, founder of the Tibetan empire, married Nepalese princess Bhrikuti Devi. As legend has it, she took paubha artists, renowned throughout Asia for the high quality of their craft, with her to Tibet.

Early paubhas were representations of religious traditions. To promote meditation, they were hung in private altars, temples, and monasteries or were carried by monks in ceremonial processions. They were rectangular, mostly uniform in size and, as they were intended for three-dimensional presentation, free of rigid frames or easels. Content, color, and proportion followed prescribed rules. Floral borders were common as was brilliant red. A main deity heavy with symbolic elements occupied the center of the composition and was surrounded by smaller less symbolic figures. The intent of the artist was iconography that would ease the path toward enlightenment. Painters for the most part remained anonymous.

Festival Banner on this month’s cover of Emerging Infectious Diseases is a scroll painting in the paubha tradition. It was likely paraded in one of the many festivals popular in Nepal to celebrate Hindu or Buddhist gods or goddesses, honor relatives, or mark the beginning or end of the agricultural cycle. The banner was part of a set depicting the Seven Mothers, important *shaktis* (feminine energies) motivating and empowering male Hindu gods. This banner shows Vaishnavi, the shakti consort of Vishnu. Also known as Adh Kanwari (eternal virgin), she attained great powers through meditation and prayer. Shaded by a multiheaded cobra hood, she traveled through the universe on the back of a mythical water monster (*makara*) (*4*).

A striking feature of Hindu and Buddhist art is the portrayal of gods with several arms and heads, symbols of multiple powers and responsibilities. Vaishnavi lifts her primary right hand in a gesture of blessing. Her remaining hands hold various symbols, among them a wheel emblazoned with the *Shri Yantra* (configuration of triangles representing the male and female principles), a lotus (sign of spiritual perfection) (*5*).

In creating sublime images of the gods, artists drew from nature. Eyes were shaped like the curve of little fish, eyebrows like an archer’s bow, lips like lotus blossoms, chin like an elephant’s trunk, female arms tapered like a root. Intricate details of lavish jewelry, headdresses, imperial robes, and garlands created a pleasing contrast against the spare stylized figure.

“The observing of figures of objects and the making of likenesses of them, which are often looked upon as idle occupation, are for a well-regulated mind a source of wisdom and an antidote against the poison of ignorance,” wrote historian and theologian Abu’Fazl Allami (1551–1602) (*6*). The unknown painters of the paubhas transformed observation of the world around them into bodies that were transcendent as well as human and created both a model and the vehicle for enlightenment.

“As the dew is dried up by the morning sun, / So are mankind’s sins at the sight of Himalaya,” read the *Puranas*. That was before global travel reached the remotest peaks and before climate change threatened the glaciers. And that was when artists painted Vaishnavi as an idealized form without evidence of muscle or bone.

Now, HIV-infected sex-trafficked women and girls from Nepal are more likely than those not infected to also have syphilis and hepatitis B (*7*). In one of her many manifestations, Vaishnavi protected her devotees from fear and gave them peace. The task now falls on global public health and its multiple hands.

## References

[1] Kramrisch S. The art of Nepal. New York: Asia Society; 1964.

[2] Brown KS. “Nepalese painting.” In: Timeline of art history. New York: The Metropolitan Museum of Art; 2000.

[3] Leoshko J, Blyth-Hill V, Goldman J. The conservation of Tibetan Thangkas: a group of symposium papers [cited 2008 Apr 17]. Available from http://palimpsest.stanford.edu/waac/wn/wn15-2/wn15-207.html

[4] From the land of the gods: Art of the Kathmandu Valley [cited 2008 Apr 17]. Available from http://www.rmanyc.org/exhibitions/nepal-mandala.xml?context=exhibitions/nepal-mandala

[5] Art of South Asia (before ca. A.D. 1500) [cited 2008 Apr 17]. Available from http://www.metmuseum.org/explore/publications/pdf

[6] Goswamy BN, Smith C. Domains of wonder: selected masterworks of Indian painting. Frome (UK): Butler and Tanner, Ltd; 2005.

[7] Silverman JG, Decker MR, Gupta JH, Dharmadhikari A, Seage GR III, Raj A. Syphilis and hepatitis B co-infection among HIV-infected, sex-trafficked women and girls, Nepal. Emerg Infect Dis. 2008;14:932–4. 18507905 10.3201/eid1406.080090 PMC2600282

